# Evaluation of Fructooligosaccharides and Inulins as Potentially Health Benefiting Food Ingredients by HPAEC-PED and MALDI-TOF MS

**DOI:** 10.1155/2009/530639

**Published:** 2009-03-18

**Authors:** Chiara Borromei, Maria Careri, Antonella Cavazza, Claudio Corradini, Lisa Elviri, Alessandro Mangia, Cristiana Merusi

**Affiliations:** Dipartimento di Chimica Generale ed Inorganica, Chimica Analitica, Chimica Fisica, Università di Parma, Viale Usberti 17/a, 43100 Parma, Italy

## Abstract

This paper describes the complementarity of high-performance anion exchange chromatography coupled with pulsed electrochemical detection (HPAEC-PED) and matrix-assisted laser desorption/ionization mass spectrometry (MALDI-TOF-MS) to evaluate commercial available fructans (fructooligosaccharides (FOS) and inulins), having different degrees of polymerization (DP) which are usually employed by food industry as functional ingredients either for their prebiotic properties or as a fat replacer, giving a fat-like mouth feel and texture. The developed HPAEC-PED methods are able to analyze FOS (fructans with DP 3–10) and inulins (DP ranging from 3 to 80) with a good resolution and relatively short retention times to evaluate structural differences between fructooligosaccharide and inulins and the possible presence of inulooligosaccharides as well as of branching. To characterize FOS and inulin at different degrees of polymerization and to assure correct molecular assignment, MALDI-TOF MS analysis was also investigated. The 2,5-dihydroxy benzoic acid (2,5-DHB) was found to be the best matrix for FOS analysis as Actilight and Raftilose P95 products, while 3-aminoquinoline (3-AQ) seems to be the best matrix for inulin with higher DP. The applicability of the optimized methods to the identification and determination of FOS contained in a symbiotic milk as well as a type of inulin added as functional ingredient to a cooked ham is demonstrated.

## 1. Introduction

Fructans are carbohydrate polymers consisting of a sucrose molecule that is elongated by a chain of fructosyl units connected through *β*-(2→1) or *β*-(2→6) linkages [1], depending on the linkage type they are called inulin and levans, respectively.

Inulin has been defined as a polydisperse carbohydrate material consisting mainly, if not exclusively, of *β*-(2→1) fructosyl-fructose linkes, containing one terminal glucose as in sucrose and having the generic chemical structure GFn (with G as glucose, F as fructose, and n indicating DP). When referring to the definition of inulin, both GFn and Fn compounds, consisting exclusively, of *β*-(2→1) fructosyl-fructose linkes, are considered to be included under this same nomenclature. Several inulin types occur in nature and they differ for the degree of polymerization and molecular weight, depending on the source, the harvest time and processing conditions [2].

Fructooligosaccharides (FOS) with DP 3–9 (average DP 4.5) are produced during the process of chemical degradation or controlled enzymatic hydrolysis of inulin by endoglycosidases [3, 4]. Furthermore, FOS can be produced on a commercial scale, from sucrose, using a fungal enzyme from either *Aureobasidium* sp. [5] or *Aspergillus niger* [6].

FOS and inulin are recognized as health-promoting food ingredients. The variety of chemical and structural conformations that characterize FOS and inulins makes them flexible and appealing ingredients for different food applications. Inulin has been reported to develop a gel-like structure when thoroughly mixed with water or other aqueous liquid, forming a gel with a white creamy appearance, which can be easily incorporated into foods to replace fats making inulin an interesting ingredient to deliver structure in low or zero fat-food products [7]. Correlation between inulin gel properties and its chemical structure (oligo- and polysaccharides) has been evaluated [8]. Furthermore, FOS and inulin exhibit prebiotic function stimulating the growth and/or activity of one or a limited number of bacteria in the colon that can improve host health [9] and, therefore, they can be employed in functional food formulations [10]. In a previous work, prebiotic effectiveness of FOS and inulin of different degrees of polymerization was reported [11], and the response of bifidobacteria to differently lengthened fructans was analyzed in pure and fecal cultures, confirming that fermentation of FOS and inulins in the colon can be correlated to different metabolic activities carried out by several intestinal microorganisms [12]. Carbohydrate analyses conducted at the end of batch fermentations by high-performance anion-exchange chromatography (HPAEC) with pulsed electrochemical detection (PED) technique demonstrated a very heterogeneous strain-dependent capability to degrade FOS or inulins. It was demonstrated that during batch fermentations, the short fructans were fermented first then gradually the longer ones were consumed. However, regarding the investigated carbohydrates, only qualitative indications were given with respect to the chain polymerization degree.

Within the panel of analytical techniques available for the characterization of fructooligosaccharides (FOS) and inulin, HPAEC-PED can be a useful and sensitive tool for the qualitative chain-length analysis of oligo- and polysaccharides from polydisperse preparations such as FOS and inulin at different degrees of polymerization [8–11]. Besides, HPAEC-PED is routinely used to separate neutral and charged oligosaccharides differing by branch, linkage, and positional isomerism [13]; from the chromatograms generated by HPAEC it is not possible to identify each observed component, without access to reference material.

The lack of standards is an obvious problem when investigating FOS and inulin contain linear homologous series of fructan polysaccharides, of which there are no commercial standards available.

Matrix-assisted laser desorption/ionization time-of-flight mass spectrometry (MALDI-TOF-MS) has been used in molecular sizing of carbohydrates and the technique can conveniently be combined with other methods such as HPLC, demonstrating to be a powerful tool for the characterization of carbohydrates [14–16]. Furthermore, MALDI-TOF-MS has been used to determine chain length distribution of FOS and inulin [17, 18] as well as for both qualitative and quantitative analyses in selected food samples [19, 20].

This paper deals with the development of HPAEC-PED methods to characterize and compare FOS and inulins having different degrees of polymerization. The qualitative HPAEC-PED profiles were then compared with molecular weight distribution evaluated by MALDI-TOF-MS. Our results could be important for labeling or supporting a prebiotic claim in food as they give indications regarding DP distribution and the amount of FOS or inulin contained. Furthermore, this work describes the validation and application of HPAEC-PED methods for the quantitative determination of short-chain FOS (sc-FOS) added to a symbiotic milk and inulin added to a cooked ham as a functional ingredient.

## 2. Materials and Methods

### 2.1. Chemicals

The deionized water (18 MΩ cm resistivity) was obtained from a Milli-Q element water purification system (Millipore, Bedford, Mass, USA). Acetonitrile, methanol (both of HPLC purity), trifluoroacetic acid, and formic acid (analytical reagent grade) were purchased from Carlo Erba (Milan, Italy). Sodium hydroxide and sodium nitrate were from J. T. Baker (Deventer, The Netherlands). Carrez reagent I (potassium hexacyanoferrate(II) trihydrate), carrez reagent II (zinc acetate), glucose, fructose, and sucrose and lactose were purchased from Sigma-Aldrich (Milan, Italy). 1-kestose was from Fluka (Milan, Italy). All sample solutions were filtered through a Type 0.45 *μ*m single-use membrane filter (Millipore, Bedford, Mass, USA).

### 2.2. HPAEC-PED Apparatus

All HPAEC-PED experiments were performed using a DX 500 system equipped with a GP40 pump, using a CarboPac PA-200 column (Dionex, 3 × 250 mm) and a CarboPac PA-100 (Dionex, 4 × 250 mm), connected to the associated guard column. Carbohydrates were detected by a model ED40 electrochemical detector in its integrated pulsed amperometric detection mode, applying the following potentials and durations: *E*_1_ = 0.10  V  (*t*_1_ = 0.40  second), *E*_2_ = −2.00  V  (*t*_2_ = 0.01  second), *E*_3_ = 0.60  V  (*t*_3_ = 0.01  second), *E*_4_ = −0.10  V  (*t*_4_ = 0.06  second). Integration is between 0.20 and 0.40 seconds. The chromatographic system was interfaced, via proprietary network chromatographic software (PeakNet TM) to a personal computer, for instrumentation control, data acquisition, and processing. All were from Dionex Corporation (Sunnyvale, Calif, USA).

### 2.3. Chromatographic Conditions

To separate FOS and inulin at different degrees of polymerization (DP), on a CarboPac PA-200 column, the mobile phase consisted of deionized water (eluent A), 600 mM aqueous sodium hydroxide (eluent B), and 250 mM aqueous sodium nitrate solution (eluent C), employing a gradient program as reported in method 1, Table 1. A similar procedure was developed to characterize the oligosaccharide distribution present in a commercial available product containing short chain fructooligosaccharides (scFOS) (Actilight 950P), in which carbohydrates were separated on the CarboPac PA100 column, eluting by the gradient reported in method 2, Table 1, in which eluent A was water, eluent B 600 mM aqueous sodium hydroxide solution, and eluent C 500 mM aqueous sodium acetate solution. The gradient elution program reported in Table 2 was applied to elute oligosaccharide fraction present in inulin added to cooked ham.

All mobile phases were sparged and pressurized with helium to prevent adsorption of atmospheric carbon dioxide and subsequent production of carbonate, which would act as displacing ion and shorten retention time.

### 2.4. MALDI-TOF-MS Analysis of FOS

MALDI-MS measurements were performed using an MALDI-LR time-of-flight mass spectrometer (Micromass, Manchester, UK) operating in the positive linear ion mode. Ions formed by a pulsed UV laser beam (*λ* = 337  nm) were accelerated at 15 keV. Laser strength was varied from sample to sample to obtain the best signal.

For all samples, different matrices were tested: 2,5-dihydroxy benzoic acid (2,5-DHB) (Sigma-Aldrich), trihydroxyacetophenone (THAP), 3-aminoquinoline (3-AQ), hydroxylphenylazo benzoic acid (HABA), and 4-hydroxy-*α*-alpha cyanocinnamic acid (HCCA) (Fluka), at 10 mg mL^−1^ in either aqueous solution and water/acetonitrile (50/50 v/v) trifluoroacetic acid (TFA) (0.1% v/v) mixture. A dried droplet sample preparation was adopted. Three replicated measurements were performed on each sample. External calibration was performed using the [M+H]^+^ ions of a peptide mixture (angiotensin I, angiotensin II, substance P, rennin, ACTH, insulin bovine, cytochrome c) (Sigma-Aldrich).

### 2.5. Samples

Different sources of fructans were analyzed both by HPAEC-PED and MALDI-TOF-MS: Raftiline ST, Raftilose P95 (Orafti, Tienen, Belgium), Actilight 950P (Beghin Mijie, Thumeries, France), Frutafit IQ, and Frutafit TEX (Sensus, Roosedaal, The Netherlands). All stock solutions were prepared at 1 mg mL^−1^ with HPLC-grade water and filtered on a 0.45 *μ*m membrane filter. Cooked ham was kindly provided by I Fratelli Emiliani SpA (Langhirano, Parma, Italy). Symbiotic milk was purchased from the local market.

### 2.6. Sample Preparation

Symbiotic milk was analyzed for the separation of individual sugars and short-chain fructooligosaccharides (scFOS). Two milliliters of milk were transferred to a 25 mL volumetric flask. The sample was diluted in approximately 10 mL ethanol-water (1 : 1, v/v) and 300 *μ*L Carrez I solution (stirred 1 minutr) and 300 *μ*L Carrez II solution (stirred 1 minute) were added at room temperature. Five milliliters of acetonitrile (HPLC-grade) were added. These reagents were used to precipitate the protein and noncarbohydrate fractions. The solution was made up to 25 mL with ethanol-water (1 : 1, v/v), then the solution was left for two hours until complete formation and precipitation of protein clot. The resulting solution diluted (1 : 1, v/v) with water, then was filtered through a filter paper and passed through a C_18_ Sep-Pak Plus cartridge Waters (Milford, Mass, USA) previously conditioned with 10 mL of methanol (HPLC-grade) and 10 mL of HPLC grade water. This filtered extract was forced through a 0.45 *μ*m nylon filter and then injected into the HPLC system.

Cooked ham samples were prepared by blending two slices (2.0 cm in width) of each ham and homogenization. Ten grams of the homogenized sample were weighed and diluted with 50 mL of HPLC grade water and stirred with a magnetic stirrer. The beaker with the sample was placed in a shaking water-bath at 80°C for 60 minutes to denature proteins. The sample was centrifuged at 7000 xg for 45 minutes at 4°C. The clarified solution was removed and an aliquot (1 mL) was diluted with 12 mL of HPLC-grade water (final dilution 1 : 60). After filtration through a 0.45 *μ*m membrane filter, sample was injected into HPLC.

## 3. Results and Discussion

In the first part of our work, the average degree of polymerization (DP) and distribution of oligo- and polysaccharides in commercial FOS and inulins having different molecular weight distribution were qualitatively evaluated by HPAEC-PED.

To develop an accurate, valid, and optimal chromatographic fingerprint for the quality evaluation of FOS and inulin at different degrees of polymerization (DP), the different HPAEC parameters including mobile phase composition, chromatographic column (CarboPac PA100 and CarboPac PA200), and flow rate of mobile phase were all examined and compared. The criterion used to evaluate the quality of a fingerprint was the number of peaks detected.

**Figure 1:**
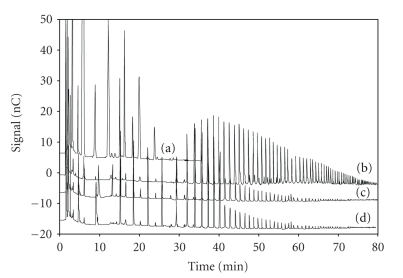
(a) Chromatographic profiles of standard solutions of Raftilose, (b) Frutafit TEX, (c) Frutafit IQ, (d) Raftiline. Chromatographic conditions as in the text.

Various eluent combinations were tested using nitrate as pushing agent to enable the selective elution with high reproducible retention time of FOS and inulin with DP up to 80 or more. Gradient elution was advantageous for separating both oligo and polymers with different DP. Under gradient conditions, acetate ion is typically the preferred “pusher” ion used by HPAEC. Using nitrate instead of acetate as the pushing agent in gradient elution of carbohydrate by HPAEC, better resolution of polymers can be achieved [21]. In our work, the dual advantages of a nitrate gradient over elution using acetate ions were the simultaneous increase of the column peak capacity and the reduction of the analysis time. Employing a CarboPac PA200 column, under the chromatographic conditions described in method 1, Table 1, both low molecular weight and high molecular weight fructans were separated (see the chromatographic profiles reported in Figure 1). The assignment of the chromatographic peaks with DP higher than 3 was based on the generally accepted assumptions that the retention time of a homologous series of carbohydrates increased as the DP increased, and that each successive peak represented a glucofructan which had a fructose more than that of the previous peak. This is because retention time increases as the number of negatively charged functional groups concurrently increases [13]. Moreover, the individual peaks were sharp and well resolved, strongly suggesting that all the analyzed samples of FOS and fructans were, as expected, mainly linear.

Instrumental precision was checked from six consecutive injections of an inulin solution; the relative standard deviations (RSDs) obtained were better than 2.7%, as reported in Table 3.

Analyzing the chromatographic profiles depicted in Figure 1, the simpler chromatographic profile was observed in sample A, which corresponds to Raftilose, where only oligosaccharides from DP 3 to DP 9 were found.

This commercially FOS product consists of a powder composed of oligosaccharide fraction including the trisaccharide 1-kestose and short-chain FOS at higher degree of polymerization as well as the natural sugars glucose, fructose, and sucrose. In chromatographic profiles depicted in Figure 1, peaks with retention times longer than 30 minutes could be assigned to polysaccharides from DP 10 to DP 80, whereas the small peaks eluting among them could correspond to isomers composed only of fructose unit chains [20], as well as slightly branched fructans [21].

Chromatographic profile of Frutafit TEX (Figure 1(b)) shows that most peaks were eluted with retention times between 32 to 80 minutes, revealing that it was mainly composed by polymeric fructans having DP higher than ten.

On the other hand, chromatographic profile depicted in Figure 1(c) was comparable to that of Figure 1(d), where a degree of polymerization number (DP) of about 80 was found for both products.

From the chromatograms generated by HPAEC, it was not possible to identify each observed component; however, by a qualitative comparison of the chromatographic profiles reported in Figure 1, it may be observed that the DP distribution of carbohydrates is very different. Frutafit IQ and Raftiline were found to be characterized by a polydisperse distribution of carbohydrates composed of both linear oligofructose (very similar to that of Raftilose, Figure 1(a)) and polifructose at higher DP, which correspond, regarding retention times, to that of Frutafit TEX.

On comparative grounds, it is evident from the separation profiles that HPAEC–PED allowed the study of a higher number of oligomers, and it is able to compare fructans with different degrees of polymerization. Furthermore, pulsed amperometric detection is highly selective and sensitive because only reactive compounds will give response and at very low concentrations.

To verify the chain length distribution of the analyzed FOS and inulin by HPAEC-PED, the same products were analyzed by MALDI-TOF MS.

As it is well known, the matrix plays a fundamental role in quality of the MALDI-TOF MS results both in terms signal-to-noise ratio and resolution. Before comparing the MALDI-TOF MS profile of the FOS investigated by LC, the effects of different matrices were tested. Various matrices, such as 2,5-DHB, 3-AQ, HCCA, and THAP, have been previously recommended for the analysis of carbohydrates. We started by testing these four different matrices.

**Figure 2:**
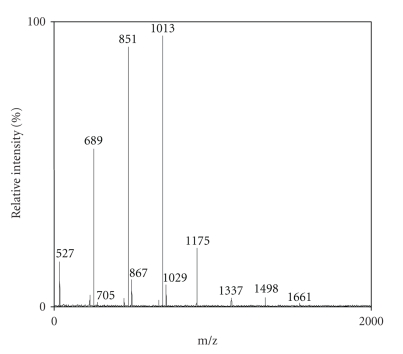
MALDI MS spectrum of a standard solution (x mg mL^−1^) of Raftilose. DHB was used as matrix. “a.i.” means arbitrary intensity and “m/z” means the mass-to-charge ratio. Other conditions as reported in Section 2.4.

**Figure 3:**
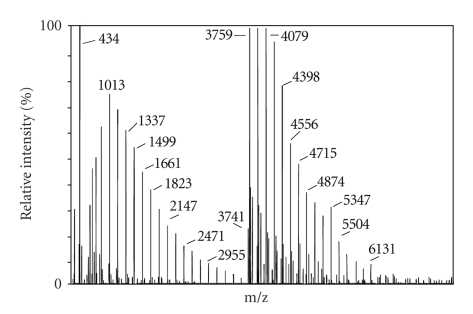
MALDI MS spectrum of a standard solution (1.0 mg mL^−1^) of Frutafit IQ. 3-AQ was used as matrix. Other conditions as reported inFigure 2.

After several experiments, DHB was found to be the best matrix for FOS analysis of Raftilose P95 (Figure 2), while 3-AQ seems to be the best matrix for inulin with a higher DP like Frutafit IQ (Figure 3) in which the spectrum was comparable with that obtained analyzing Raftiline inulin sample (data not shown).

**Figure 4:**
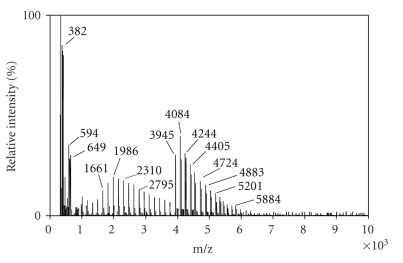
MALDI MS spectrum of a standard solution (1.0 mg mL^−1^) of Frutafit TEX. THAP was used as matrix. Other conditions as reported in Figure 2.

As reported in Figure 4, for a inulin mainly composed of fructans at high DP, such as Frutafit TEX, the best matrix resulted to be THAP with fast evaporation technique.

The MALDI-TOF mass spectra exhibited the sodium and potassium adducts and ascribed the degree of polymerization of these fructans (Table 4). The oligomers showed the mass difference of 162 Da, which corresponds to hexose residues and, as expected, all spectra exhibited the monomodal mass distributions without any fragmentation [18].

MALDI-TOF spectra elicited the same differences of DP and geometrical profiles between fructans, which were seen with HPAEC-PED.

In MALDI-TOF spectra, there are not only visibles Raftiline and Frutafit TEX Gaussian profiles like in chromatogram but also differences between Raftilose maximum relative intensity (in the ranges between DP 4–6) and Frutafit TEX maximum relative intensity (in the ranges between DP 12–14).

Many papers in literature cited the use of MALDI-TOF to analyze fructans present in natural sources as Jerusalem artichoke, onion, shallots, elephant garlic, and so forth [17–20]. MALDI-TOF MS gives better results for this type of food rather than HPAEC-PAD because fructans profiles are unknown and so MALDI-TOF MS identifications need less time to optimize analysis and assure correct molecular assignment.

Furthermore, MALDI-TOF MS is far less prone to contaminant influence and does not require a tedious purification of the analytes (which may cause selective losses of some compounds of the mixture to be analyzed) [8].

**Figure 5:**
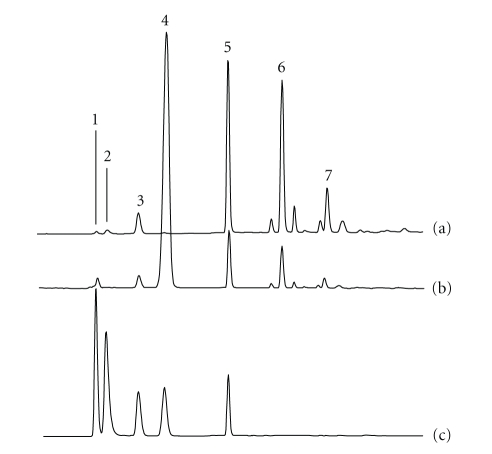
Chromatographic profiles of (a) symbiotic milk containing Actilight as prebiotic scFOS; (b) synthetic mixture of glucose, fructose, sucrose, lactose, and 1-kestose (GF3); (c) Actilight. Chromatographic conditions as reported in the text. Peak identification: (1) glucose, (2) fructose, (3) sucrose, (4) lactose, (5) 1-kestose, (6) GF4, (7) GF5.

The limit of MALDI-TOF MS is the fact that similar mass branched and linear isomers cannot be distinguished. On the other hand, although HPAEC-PED does not allow for structure elucidation, it permits identification of unknown carbohydrates relative to standards whose retention behavior versus structure has been already established. A representative elution pattern of Actilight 950P, obtained by HPAEC-PED under the chromatographic conditions reported in the experimental part, is depicted in Figure 5. Actilight, which is industrially produced through fructosyl-transfer from sucrose using a fungal enzyme, is a commercial available food ingredient, having the following composition of dry substance: 0.3% fructose, 0.4% glucose, 3.0% sucrose, 36% 1-kestose (GF2), 49% nystose (GF3), and 12% fructosyl-nystose (GF4) [22]. From the chromatographic profile A depicted in Figure 5, it is possible to clearly identify the free monosaccharide glucose and fructose, the unreacted disaccharide sucrose, and the trisaccharide 1-kestose, which were identified eluting the corresponding standards. Other oligosaccharides were selectively eluted, but the identification of the individual oligosaccharides was a challenging task due to lack of suitable standards. However, the retention times of carbohydrates from the HPAEC column depend both on DP and structural differences (e.g., branching) and evaluating the chromatographic profiles of Figure 5, we can concluded that peak no. 6 and peak no. 7 can be assigned as the tetrasaccharide nystose (GF3) and the pentasaccharide 1^F^-fructosyfuranosyl-nystose (GF4), respectively.

Moreover, the chromatographic profile of the commercial scFOS preparation shows at least other four unknown oligomers that should be assumed as inulooligosaccharides ranging from F2 to F5 [23].

The unit chain length distribution of scFOS in the Actilight product was analyzed by MALDI-TOF MS, and the obtained spectrum confirmed that the analyzed product was a mixture of short-chain oligosaccharides ranging from DP 3 to DP 5, having a mass difference of 162 Da (results not shown). Although, a distinction of oligomers having similar masses branched and linear isomers is not possible by MALDI—TOF—MS, the use of this mass spectrometric technique was a very useful tool for the molecular weight measurement, whereas the heterogeneity of scFOS distribution in Actilight was confirmed through the chromatographic profile obtained by HPAEC-PED.

### 3.1. Food Applications

#### 3.1.1. Symbiotic Milk

According to a widely accepted definition, a functional food is any modified food that has special effects on the human organism beyond the nutrients it contains [24].

Milk with added functional ingredients, but not fermented, is proposed in the market of functional foods. In these functional dairy, products are included unfermented milk with added probiotic and prebiotics, called symbiotic, due to their symbiotic functional action.

A representative pattern of a carbohydrate elution profile of commercial symbiotic functional milk is depicted by the chromatographic profile B of Figure 5. The main peaks in the chromatogram obtained on the CarboPac PA 100 column, using the elution program reported in method 2, Table 1, consist of glucose, sucrose, lactose, and scFOS. Besides the identified compounds, as reported in the chromatographic profile A, the chromatographic profile B shows the presence of minor peaks which can be identified as previously reported. Chromatographic profile C is referred to a standard solution of glucose, fructose, sucrose, lactose, and 1-kestose. The validation process of the optimized HPLC-PED method was carried out following the EURACHEM guidelines [25].

Table 5 summarizes the precision of retention times observed upon injecting the same sample of milk and using the optimized experimental conditions. The instrumental precision was evaluated by repeating the analysis of the same milk sample six times. As can be seen, relative standard deviations (RSDs) of retention times were lower than 2.10% (*n* = 6) for the same day, while these values increase up to 2.55% when the same experiment was repeated in six different days (*n* = 24). Furthermore, method repeatability was evaluated using the same data obtained for the accuracy study, where the RSDs of peak areas were in all experiments better than 2.85%.

Quantification was based on external standard method. The assay linearity was determined by the analysis of six different concentrations of the standard solutions. Each level of concentration was prepared in triplicate. The linearity of response for the analyzed sugars was demonstrated at six different concentrations from 50 to 300 *μ*g/mL for glucose, fructose, sucrose, and 1-kestose and from 50 to 450 *μ*g/mL for lactose, respectively. The standard curves were obtained by plotting peak area (*y*) versus nominal concentration *x* (*μ*gmL^−1^) of each compound and were fitted to the linear regression. The standard deviation (SD) of slope and intercept was estimated at the 95% confidence level.

The limit of detection (LOD) was defined as three times the standard deviation of the blank values (*S*_b_) divided by the slope of the calibration curves, whereas the limit of quantification (LOQ) was defined as 10 *S*_b_ divided by the slope of the calibration curve. LOD for all analyzed samples was ranging from 10.0 to 12.5 *μ*g/mL and LOQ from 25.0 to 32.0 *μ*g/mL, respectively.

Quantification of scFOS was determined from peak area using 1-kestose as the external standard. As commercial GF4 and GF5 standards were not available, they were not quantified and Actilight was quantified considering that 1-kestose represents the 36% of the whole product. The recoveries, measured at three concentration levels, varied from 97.0 to 104.3%. The validated method was successfully applied to the simultaneously determination of glucose, fructose, sucrose, lactose, and scFOS in symbiotic milk, obtaining the following results: glucose 0.58 (±0.08) mg/mL, sucrose 0.62 (±0.07) mg/mL, lactose 44.76 (±0.52) mg/mL, 1-kestose 7.28 (±0.11) mg/mL. Nystose (GF3) 78.7 mg/mL, fructofuranosylnistose (GF4) 56.8 mg/mL, and GF5 12.5 mg/mL. From the above results and data reported in literature, the content of scFOS in the examined symbiotic milk can be evaluated to be within 2.0 (±0.2)% (w/v). Furthermore, no variation of peak area ratio of 1-kestose-GF3 and 1-kestose-GF4 was noticed over ten days, demonstrating that scFOS composition did not change over the whole shelf life of the product.

#### 3.1.2. Cooked Ham

**Figure 6:**
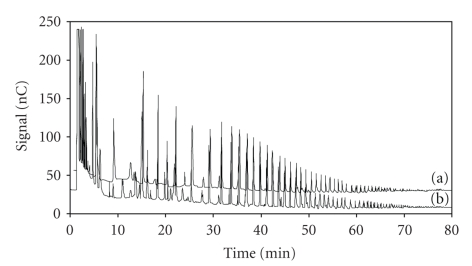
Chromatographic profiles of a standard solution (0.5 mg mL^−1^) of (a) Frutafit IQ compared with (b) the chromatographic profile of an extract of inulin from a cooked ham sample. Chromatographic conditions as in the text.

Inulin was added to the brine used in the industrial process for preparation of commercial cooked ham, prior the cooking step, as a replacer of sugars with the aim of reducing caloric content. The inulin profile of ham was analyzed by the HPAEC PED method described in method 1, Table 1. Comparing chromatographic profiles reported in Figure 6, there were no observable differences in the inulin profiles between those extracted from (b) the cooked ham and (a) the control, corresponding to inulin which was added to the brine solution. Furthermore, the same chromatographic profiles were also observed during the whole shelf life of the product (data not shown).

**Figure 7:**
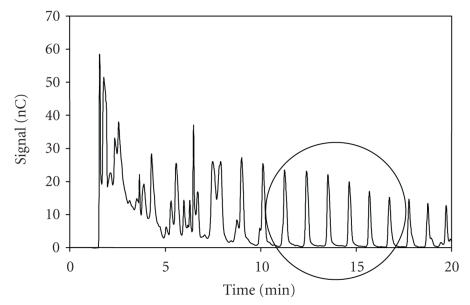
Chromatographic profile of an extract of inulin from a cooked ham sample, showing inside the circle the oligosaccharide fraction selected to perform quantitative evaluation of inulin present in the analyzed sample. Chromatographic conditions as in the text.

To perform quantitative evaluation of inulin present in cooked ham, we selected six unidentified peaks of the oligosaccharide fraction, which were selectively eluted within 20 minutes using the gradient elution program reported in Table 2 (see Figure 7). To verify the quality and usefulness of the method, the analytical parameters linearity, sensitivity, precision, and percentage of recovery were determined. The linearity of response for the selected unidentified peaks was demonstrated at six different concentrations of inulin, ranging from 50 to 300 *μ*g/mL. Higher concentrations were not assayed because we considered that the range was wide enough for the proposed applications. The linearity of the present method for all unidentified oligosaccharides selected as reference peaks was good, with correlation coefficients higher than 0.997. The limit of detection was evaluated for each of the eluted peaks, and results are summarized in Table 6. The recoveries, measured at three concentration levels of inulin added to the ham sample after homogenization, varied from 91 to 106%.

Result regarding quantitative determination of inulin in cooked ham gave results ranging from 3.20 ± 0.20 to 3.00 ± 0.42, dry matter and on the average 1.05 (±0.02)g on 100 g of cooked ham.

## 4. Conclusions

We have demonstrated the usefulness of MALDI-TOF MS, and that high-pH anion exchange chromatography with pulsed amperometric detection (HPAEC-PAD) provides powerful tools for the analysis of FOS and inulins with a high sensitivity and no need for derivatization.

HPAEC-PED methods were developed to determine chain length distribution of FOS and inulins, which were compared with the corresponding distributions from MALDI-TOF MS analyses. Although some differences were observed, the developed HPAEC-PED methods can be considered as secondary or orthogonal methods complementary to MALDI-TOF MS to evaluate oligo- and polysaccharides distribution in the analyzed samples. MALDI-MS gives better assurance of correct molecular assignment since the isotopic mass of each peak is available, although similar masses branched and linear isomers cannot be distinguished.

By using HPAEC and MALDI-TOF MS analysis, the presence of FOS and inulin at different degrees of polymerization could neither be demonstrated in ingredient preparations nor from functionalized foods.

## Figures and Tables

**Table 1:** Gradient elution program to elute (method 1) FOS and inulin and (method 2) scFOS.

HPAEC-PED method 1					HPAEC-PED method 2				
Elution time (min)	A(%)	B(%)	C^1^(%)	Comment	Elution time (min)	A(%)	B(%)	C^2^(%)	Comment
−40^a^	0	100	0	Start cleaning step	−45^a^	0	100	0	Start cleaning step
−30^a^	0	100	0	End cleaning step	−30^a^	0	100	0	End cleaning step
−29.9^a^	89	10	1	Start conditioning step	−29.9^a^	89	10	1	Start conditioning step
0	89	10	1	End conditioning step	0	89	10	1	End conditioning step
0.1	89	10	1	Injection, acquisition start	0.1	89	10	1	Injection, acquisition start
4	84	15	1	End first gradient step	5	89	10	1	End isocratic elution
					45	60	20	20	End first gradient step
80	79	15	40	End third gradient step	55	50	20	30	End second gradient step

A: deionized water; B: sodium hydroxide (600 mM); C^1^: sodium nitrate (250 mM); C^2^: sodium acetate (500 mM).

^a^Negative time indicates time prior to sample injection.

**Table 2:** Gradient elution program toelute oligosaccharide fraction present in inulin added to cooked ham.

Elution time (min)	A(%)	B(%)	C^1^(%)	Comment
−35^a^	0	100	0	Start cleaning step
−25^a^	0	100	0	End cleaning step
−24.9^a^	79	16	5	Start conditioning step
0	79	16	5	End conditioning step
0.1	74	16	10	Injection, acquisition start
20	62	16	22	End first gradient step

A: deionized water; B: sodium hydroxide (600 mM); C: sodium acetate (500 mM).

^a^Negative time indicates time prior to sample injection.

**Table 3:** Repeatability of retention time (sample Frutafit IQ).

N.Peaks	Retention time ±SD	cv(%)	N.Peaks	Retention time ±SD	cv(%)
1	1.90 ± 0.04	0.92	44	48.09 ± 0.32	0.40
2	1.97 ± 0.57	1.52	45	49.01 ± 0.41	0.33
3	2.10 ± 0.09	2.50	46	49.18 ± 0.38	0.48
4	2.60 ± 0.92	2.06	47	50.09 ± 0.40	0.46
5	3.12 ± 1.52	2.72	48	50.28 ± 0.33	0.47
6	4.70 ± 2.50	2.47	49	51.11 ± 0.48	0.45
7	5.73 ± 2.06	2.03	50	51.30 ± 0.46	0.35
8	9.11 ± 2.72	1.94	51	52.14 ± 0.47	0.33
9	12.38 ± 2.47	1.75	52	52.36 ± 0.45	0.30
10	15.15 ± 2.03	1.91	53	53.10 ± 0.35	0.30
11	16.38 ± 1.94	2.45	54	53.34 ± 0.33	0.32
12	18.82 ± 1.75	2.55	55	54.03 ± 0.30	0.32
13	20.36 ± 1.91	2.59	56	54.28 ± 0.30	0.25
14	22.64 ± 2.45	2.62	57	54.96 ± 0.32	0.23
15	24.52 ± 2.55	2.40	58	55.24 ± 0.32	0.21
16	26.40 ± 2.59	1.65	59	55.83 ± 0.25	0.10
17	27.00 ± 2.62	1.02	60	56.09 ± 0.23	0.14
18	28.50 ± 2.40	1.12	61	56.65 ± 0.21	0.04
19	29.92 ± 1.65	0.95	62	56.92 ± 0.10	0.41
20	31.26 ± 1.02	0.80	63	57.49 ± 0.14	0.52
21	31.55 ± 1.12	0.72	64	57.75 ± 0.04	0.62
22	32.37 ± 0.95	0.63	65	58.30 ± 0.41	0.51
23	33.79 ± 0.80	0.62	66	58.65 ± 0.52	0.45
24	34.31 ± 0.72	0.57	67	59.22 ± 0.62	0.39
25	35.64 ± 0.63	0.53	68	59.63 ± 0.51	0.35
26	35.99 ± 0.62	0.51	69	60.19 ± 0.45	0.33
27	37.33 ± 0.57	0.48	70	60.55 ± 0.39	0.29
28	37.57 ± 0.53	0.26	71	61.10 ± 0.35	0.04
29	38.89 ± 0.51	0.35	72	61.51 ± 0.33	0.24
30	39.05 ± 0.48	0.31	73	61.96 ± 0.29	0.28
31	40.26 ± 0.26	0.31	74	62.42 ± 0.04	0.20
32	40.43 ± 0.35	0.25	75	62.76 ± 0.24	0.15
33	41.62 ± 0.31	0.19	76	63.07 ± 0.28	0.29
34	41.76 ± 0.31	0.51	77	63.49 ± 0.20	0.27
35	43.04 ± 0.25	0.56	78	63.88 ± 0.15	0.36
36	43.21 ± 0.19	0.31	79	64.13 ± 0.29	0.43
37	44.21 ± 0.51	0.51	80	64.44 ± 0.27	0.47
38	44.37 ± 0.56	0.35	81	64.75 ± 0.36	0.48
39	45.52 ± 0.31	0.58	82	65.03 ± 0.40	0.45
40	45.78 ± 0.51	0.39	83	65.33 ± 0.47	0.43
41	46.72 ± 0.35	0.32	84	65.64 ± 0.48	0.35
42	47.01 ± 0.58	0.41	85	65.94 ± 0.45	0.37
43	47.87 ± 0.39	0.38	86	66.20 ± 0.43	0.35

**Table 4:** Example of sodium adduct and degree of polymerization of Raftiline standard.

Degree of polymerization (DP)	[M+Na]^+^	Degree of polymerization (DP)	[M+Na]^+^
DP3	527	DP17	2799.3
DP4	689	DP18	2961.6
DP5	852.1	DP19	3124
DP6	1014.6	DP20	3286.1
DP7	1176.9	DP21	3448.5
DP8	1339.4	DP22	3610.1
DP9	1501.7	DP23	3772.7
DP10	1664	DP24	3934.4
DP11	1826.3	DP25	4096.4
DP12	1988.5	DP26	4258.4
DP13	2150.9	DP27	4420.9
DP14	2313.1	DP28	4582.7
DP15	2475.3	DP29	4743.7
DP16	2637.4	DP30	4905.3

**Table 5:** Reproducibility of retention times for intra- and interday analyses for the symbiotic milk sample by HPAEC-PED (separation conditions as in Figure 5).

	Intraday (*n* = 6)		Interday (6 days, *n* = 24)	
Compound	Retention time (min)	RSD (%)	Retention time (min)	RSD (%)
Glucose	5.75	1.88	5.93	2.24
Fructose	6.87	2.08	7.05	2.75
Saccharose	8.90	1.91	10.10	2.60
Lactose	12.41	1.82	12.62	2.44
1-kestose	15.90	1.77	16.10	2.10

**Table 6:** Limits of detection and quantitation.

Peaks	1	2	3	4	5	6
Limit of detection (*y*D)^a^ *μ*g/g	12.21	13.65	12.45	13.22	14.27	16.03
Limit of quantification (yQ)^b^ *μ*g/g	43.32	48.62	49.89	40.67	50.77	51.21

^a^ Concentration corresponding to signal andyD = yb + 2*t*(95%, *n* − 1)sb.

^b^ Concentration corresponding to signal and yQ = yb + 10sb.

## Nomenclature

HPAEC-PED: High performance anion exchange chromatography with pulsed electrochemical detection
MALDI-TOF-MS: Matrix-assisted laser desorption/ionization time-of-flight mass spectrometry
MALDI-LR: Linear mode
DP: Degrees of polymerization
FOS: Fructooligosaccharides
DHB: Dihydroxybenzoic acid
THAP: Trihydroxyacetophenone
AQ: Aminoquinoline
HCCA: Hydroxy-*α*-alpha cyanocinnamic acid
TFA: Trifluoroacetic acid
ACTH: Adrenocorticotrophic hormone.

## References

[1] Ernst MK, Chatterton NJ, Harrison PA, Matitschka G (1998). Characterization of fructan oligomers from species of the genus *Allium* L. *Journal of Plant Physiology*.

[2] Vijn I, Smeekens S (1999). Fructan: more than a reserve carbohydrate?. *Plant Physiology*.

[3] Crittenden RG, Playne MJ (1996). Production, properties and applications of food-grade oligosaccharides. *Trends in Food Science & Technology*.

[4] Roberfroid MB, Van Loo JAE, Gibson GR (1998). The bifidogenic nature of chicory inulin and its hydrolysis products. *The Journal of Nutrition*.

[5] Yun JW (1996). Fructooligosaccharides—occurrence, preparation, and application. *Enzyme and Microbial Technology*.

[6] Hidaka H, Eida T, Takizawa T, Tokunaga T, Tashiro Y (1986). Effects of fructooligosaccharides on intestinal flora and human health. *Bifidobacteria Microflora*.

[7] Teeuwen H, Thonè M, Vandorpe J (1992). Inulin: a versatile fibre ingredient. *International Food Ingredients*.

[8] Chiavaro E, Vittadini E, Corradini C (2007). Physicochemical characterization and stability of inulin gels. *European Food Research and Technology*.

[9] Gibson GR, Roberfroid MB (1995). Dietary modulation of the human colonic microbiota: introducing the concept of prebiotics. *The Journal of Nutrition*.

[10] Huebner J, Wehling RL, Hutkins RW (2007). Functional activity of commercial prebiotics. *International Dairy Journal*.

[11] Corradini C, Bianchi F, Matteuzzi D, Amaretti A, Rossi M, Zanoni S (2004). High-performance anion-exchange chromatography coupled with pulsed amperometric detection and capillary zone electrophoresis with indirect ultra violet detection as powerful tools to evaluate prebiotic properties of fructooligosaccharides and inulin. *Journal of Chromatography A*.

[12] Rossi M, Corradini C, Amaretti A (2005). Fermentation of fructooligosaccharides and inulin by bifidobacteria: a comparative study of pure and fecal cultures. *Applied and Environmental Microbiology*.

[13] Lee YC (1996). Carbohydrate analyses with high-performance anion-exchange chromatography. *Journal of Chromatography A*.

[14] Mock KK, Daevy M, Cottrell JS (1991). The analysis of underivatized oligosaccharides by matrix-assisted laser desorption mass spectrometry. *Biochemical and Biophysical Research Communications*.

[15] Jiang G, Vasanthan T (2000). MALDI-MS and HPLC quantification of oligosaccharides of lichenase-hydrolyzed water-soluble *β*-glucan from ten barley varieties. *Journal of Agricultural and Food Chemistry*.

[16] Harvey DJ (2003). Matrix-assisted laser desorption/ionization mass spectrometry of carbohydrates and glycoconjugates. *International Journal of Mass Spectrometry*.

[17] Stahl B, Linos A, Karas M, Hillenkamp F, Steup M (1997). Analysis of fructans from higher plants by matrix-assisted laser desorption/ionization mass spectrometry. *Analytical Biochemistry*.

[18] Štikarovská M, Chmelík J (2004). Determination of neutral oligosaccharides in vegetables by matrix-assisted laser desorption/ionization mass spectrometry. *Analytica Chimica Acta*.

[19] Wang J, Sporns P, Low NH (1999). Analysis of food oligosaccharides using MALDI-MS: quantification of fructooligosaccharides. *Journal of Agricultural and Food Chemistry*.

[20] Štikarovská M, Chmelík J (2004). Determination of neutral oligosaccharides in vegetables by matrix-assisted laser desorption/ionization mass spectrometry. *Analytica Chimica Acta*.

[21] Zhang Y, Inoue Y, Inoue S, Lee YC (1997). Separation of oligo/polymers of 5-*N*-acetylneuraminic acid, 5-*N*-glycolylneuraminic acid, and 2-keto-3-deoxy-D-glycero-D-galacto-nononic acid by high-performance anion-exchange chromatography with pulsed amperometric detector. *Analytical Biochemistry*.

[22] Król B, Grzelak K (2006). Qualitative and quantitative composition of fructooligosaccharides in bread. *European Food Research and Technology*.

[23] Ronkart SN, Blecker CS, Fourmanoir H (2007). Isolation and identification of inulooligosaccharides resulting from inulin hydrolysis. *Analytica Chimica Acta*.

[24] Diplock AT, Aggett PJ, Ashwell M, Bornet F, Fern EB, Roberfroid MB (1999). Scientific concepts of functional foods in Europe: consensus document. *British Journal of Nutrition*.

[25] The Fitness for Purpose of Analytical Methods: A Laboratory Guide to Method Validation and Related. http://www.eurachem.ul.pt.

